# Differences in rectal amino acid levels determine bacteria-originated sex pheromone specificity in two closely related flies

**DOI:** 10.1038/s41396-023-01488-9

**Published:** 2023-08-07

**Authors:** Zijie Gao, Mingxue Xie, Shiyu Gui, Muyang He, Yongyue Lu, Luoluo Wang, Jingyuan Chen, Guy Smagghe, Jonathan Gershenzon, Daifeng Cheng

**Affiliations:** 1 Department of Entomology, South China Agricultural University, Guangzhou, 510640 China; 2 Advanced Institute of Natural Sciences, Beijing Normal University, Zhuhai, 519087 China; 3 Department of Plants and Crops, Faculty of Bioscience Engineering, Ghent University, Ghent, 9000 Belgium; 4 Institute of Entomology, Guizhou University, Guiyang, 550025 China; 5 Molecular and Cellular Life Sciences, Department of Biology, Vrije Universiteit Brussel (VUB), Brussels, 1050 Belgium; 6 Department of Biochemistry, Max Planck Institute for Chemical Ecology, Jena, 07745 Germany

**Keywords:** Molecular ecology, Animal physiology

## Abstract

Sex pheromones are widely used by insects as a reproductive isolating mechanism to attract conspecifics and repel heterospecifics. Although researchers have obtained extensive knowledge about sex pheromones, little is known about the differentiation mechanism of sex pheromones in closely related species. Using *Bactrocera dorsalis* and *Bactrocera cucurbitae* as the study model, we investigated how the male-borne sex pheromones are different. The results demonstrated that both 2,3,5-trimethylpyrazine (TMP) and 2,3,5,6-tetramethylpyrazine (TTMP) were sex pheromones produced by rectal *Bacillus* in the two flies. However, the TMP/TTMP ratios were reversed, indicating sex pheromone specificity in the two flies. Bacterial fermentation results showed that different threonine and glycine levels were responsible for the preference of rectal *Bacillus* to produce TMP or TTMP. Accordingly, threonine (glycine) levels and the expression of the threonine and glycine coding genes were significantly different between *B. dorsalis* and *B. cucurbitae*. In vivo assays confirmed that increased rectal glycine and threonine levels by amino acid feeding could significantly decrease the TMP/TTMP ratios and result in significantly decreased mating abilities in the studied flies. Meanwhile, decreased rectal glycine and threonine levels due to RNAi of the glycine and threonine coding genes was found to significantly increase the TMP/TTMP ratios and result in significantly decreased mating abilities. The study contributes to the new insight that insects and their symbionts can jointly regulate sex pheromone specificity in insects, and in turn, this helps us to better understand how the evolution of chemical communication affects speciation.

## Introduction

Pheromones are substances secreted by one individual into the body and detected by other individuals of the same species through the olfactory organs, causing the latter to exhibit certain behavioral, emotional, psychological or physiological changes [1]. Pheromones have a communication function, and almost all animals have demonstrated the presence of pheromones [1]. In a variety of animal species, sex pheromones are used for the attraction and selection of potential mates. In many insect species, sex pheromones are important species-recognition signals [2]. Sex pheromones are considered to have evolved due to interactions between species since they have dual roles in attracting conspecifics and inhibiting interspecific attraction [3]. Studies have shown that variations in sex pheromones exist even within populations. Sex pheromones can be plastic in different populations, and this plasticity can be translated into quantitative [4, 5] or qualitative differences [6]. Such changes can even take place over an individual’s lifespan [7].

The traditional view is that variations in sex pheromone composition can lead to reproductive isolation between closely related species, which is a driving force of speciation [8]. Closely related species usually have similar sex pheromones that may only differ in the ratio of the different chemical components. Small changes in the ratio of chemical components in pheromones can affect attraction. Due to the complex interaction between interspecific selection forces, more integrative studies on the evolution of sex pheromone communication are needed.

The family Tephritidae consists of more than 4000 species, many of which extensively affect fruit or vegetable production throughout the world [9]. Adult female flies can cause direct damage by laying eggs in fruits or vegetables. For tephritid flies, courtship and mating can be influenced by both physical and olfactory cues. It is generally acknowledged that tephritid males produce sex pheromones to attract females. In recent decades, studies have indicated that two typical tephritid species (*Bactrocera cucurbitae* and *Bactrocera dorsalis*) produce volatile compounds that are used as sex pheromones. It has been observed that *B. cucurbitae* males produce a pheromone substance that is visible as “smoke” at dusk to attract virgin females [10]. Moreover, researchers have indicated that pyrazines released from the excised rectal glands of males are able to elicit responses from female flies [11]. The oriental fruit fly *B. dorsalis* is another peculiar tephritid species. *B. dorsalis* males can also release molecules to attract female flies. Studies have indicated that pyrazines are also produced in the rectal glands of males [12], and our previous studies have indicated that these volatiles originate from bacteria and play roles as sex pheromones [13].

In this study, we investigated the generation of sex pheromones (pyrazines) in both *B. cucurbitae* and *B. dorsalis* and compared sex pheromone specificity between the two species. The results indicated that rectal bacteria are responsible for producing sex pheromones in the rectum of males of both species. However, the ratios of pyrazines in different species were significantly different, and different rectal threonine and glycine levels regulated by the glycine and threonine metabolism pathway in the rectum were responsible for such differences.

## Results

### Different TMP/TTMP ratios suggest sex pheromone specificity between *B. dorsalis* and *B. cucurbitae*

**Fig. 1:**
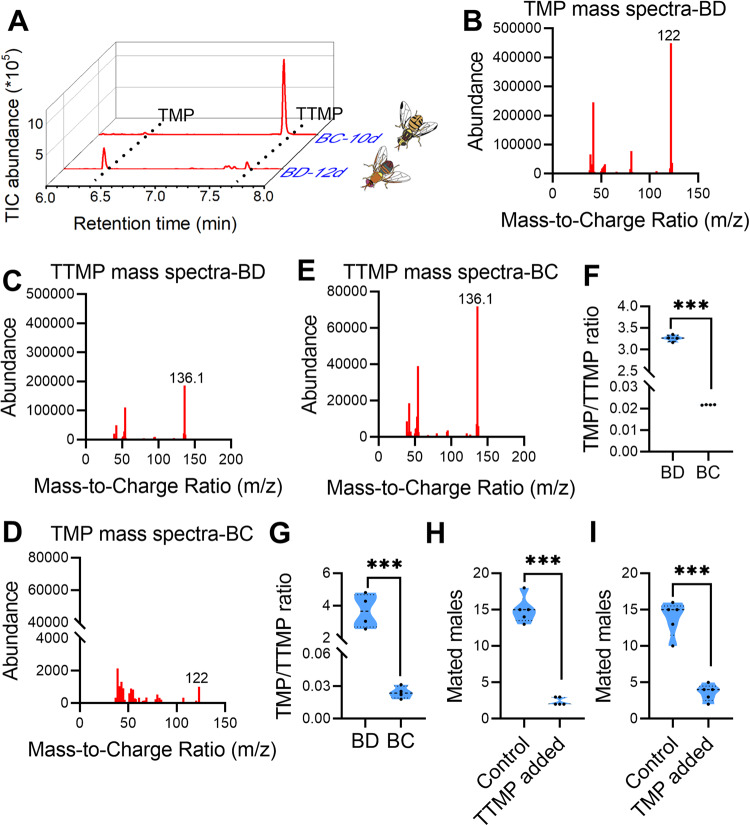
Sex pheromone identification in *B. dorsalis* (BD) and *B. cucurbitae* (BC). **A** GC‒MS ion chromatograms of mature male rectum extracts of BD and BC. Traces for the flies expressing TMP and TTMP are shown with dotted lines. **B**, **C** GC‒MS mass spectra intensity of TMP and TTMP in BD. **D**, **E** GC‒MS mass spectra intensity of TMP and TTMP in BC. **F** TMP/TTMP ratio comparison between BD and BC (*n* = 4 replicates, independent sample *t* test, ***p < 0.001). **G** TMP/TTMP ratio comparison between wild populations of BD and BC (*n* = 4 replicates, independent sample *t* test, ***p = 0.001). **H** Mating ability comparisons between BD males added TTMP and the control (*n* = 5 replicates, paired sample t test, *t* = 26.87, ***p < 0.001). **I** Mating ability comparisons between BC males added TMP and the control (*n* = 5 replicates, paired sample t test, *t* = 17.25, ***p < 0.001).

The volatile compounds in the mature male rectum extracts of *B. dorsalis* and *B. cucurbitae* were analyzed with gas chromatography–mass spectrometry (GC‒MS). The results indicated that the major products with the same retention time were detected in both *B. dorsalis* and *B. cucurbitae* male rectums (Fig. 1A). These products were tentatively identified as TMP (characteristic fragment ions: 122) and TTMP (characteristic fragment ions: 136.1) based on their mass spectra (Fig. 1B–E). Interestingly, the TMP/TTMP ratios were completely reversed between *B. dorsalis* and *B. cucurbitae*, and the TMP/TTMP ratios were significantly higher in *B. dorsalis* than in *B. cucurbitae* (Fig. 1F). Since our previous study had shown a TMP/TTMP ratio of around 1:1 in *B. dorsalis* male rectums [13], we further examined the ratios for wild populations of *B. dorsalis* and *B. cucurbitae* to verify the results. The results showed that TMP/TTMP ratios were also reversed between wild populations of *B. dorsalis* and *B. cucurbitae* (Fig. 1G and Supplementary Fig. S1). Given that TMP and TTMP could only be detected in mature male rectum extracts of *B. dorsalis* (12-day-old) and *B. cucurbitae* (10-day-old) (Supplementary Fig. S1) and only mature male rectum extracts attracted the mature female (Supplementary Fig. S2), we reason that TMP and TTMP are sex pheromones of *B. dorsalis* and *B. cucurbitae* males. To verify if sex pheromone specificity between *B. dorsalis* and *B. cucurbitae* is conferred by different TMP/TTMP ratios, attraction to females and mating abilities of males added with TMP or TTMP solutions were tested. The results indicated that TMP/TTMP ratios decreased (or increased) in males of *B. dorsalis* (or *B. cucurbitae*) significantly decreased the attraction to females and thus reducing the mating ability of the males (Fig. 1H, I). These results show that the different TMP/TTMP ratios may suggest sex pheromone specificity between *B. dorsalis* and *B. cucurbitae*.

### Rectal bacterial differences between *B. dorsalis* and *B. cucurbitae* are not associated with different TMP/TTMP ratios

**Fig. 2:**
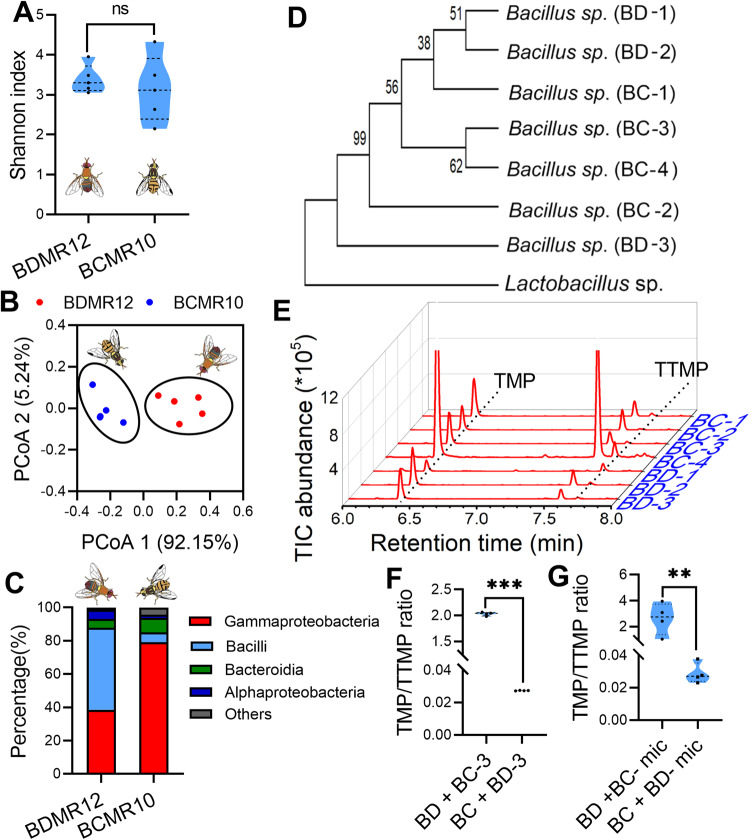
Rectal bacteria difference comparison between *B. dorsalis* (BD) and *B. cucurbitae* (BC). **A** Violin plots showing the estimated richness of the microbial community based on Shannon indices of the 16S amplicon sequences (*n* = 5 replicates, independent sample *t* test, “ns” no significance). **B** Principal coordinate analysis of the microbial community structure in *B. dorsalis* and *B. cucurbitae* (beta diversity, class level) measured by the Bray‒Curtis distance matrix of 16S rRNA gene amplicon sequences. BDMR12: 12-day-old (mature) rectum of *B. dorsalis*, BCMR10: 10-day-old (mature) rectum of *B. cucurbitae*. **C** Class-level relative abundance of 16S rRNA gene amplicon sequences. Values are averaged according to *B. dorsalis* and *B. cucurbitae* mature male rectums. **D** Phylogenetic analysis of *Bacillus* isolated from *B. dorsalis* and *B. cucurbitae*. A maximum likelihood phylogeny is presented with bootstrap values (500 times). **E** GC‒MS ion chromatograms of volatiles produced by *Bacillus* isolated from the male rectum of *B. dorsalis* and *B. cucurbitae*. Traces for TMP and TTMP are shown with dotted lines. **F** TMP/TTMP ratio comparison between *B. dorsalis* fed *Bacillus* of *B. cucurbitae* and *B. cucurbitae* fed *Bacillus* of *B. dorsalis* (*n* = 4 replicates, independent sample *t* test, ***p < 0.001). **G** TMP/TTMP ratio comparison between *B. dorsalis* fed microbiota of *B. cucurbitae* and *B. cucurbitae* fed microbiota of *B. dorsalis* (*n* = 4 replicates, independent sample *t* test, * *p* = 0.043).

Previous studies have indicated that *Bacillus* can produce TMP and TTMP [14–16], and our previous study has shown that TMP and TTMP production in the rectum of *B. dorsalis* is associated with rectal *Bacillus* [13]. Thus, a plausible assumption is that the pheromone synthesis mechanism is conserved in the two studied flies, and the difference in the metabolic efficiency of *Bacillus* may be the cause of the different TMP/TTMP ratios between *B. dorsalis* and *B. cucurbitae*. To verify this hypothesis, the abundance and composition of the rectum microbial communities was inferred by 16S ribosomal RNA gene amplicon sequencing (Supplementary Dataset S1). Alpha diversity in 16S rRNA amplicon sequencing indicated that rectal bacterial community diversity was not significantly different between *B. dorsalis* and *B. cucurbitae* (Fig. 2A, Supplementary Dataset S2). However, the Bray‒Curtis beta diversity shown by principal coordinate analysis (PCoA) was significantly different between *B. dorsalis* and *B. cucurbitae* (Fig. 2B), and the key class, Bacilli, was more highly enriched in *B. dorsalis* than in *B. cucurbitae* (Fig. 2C). To further verify whether rectal *Bacillus* species were responsible for the different TMP/TTMP ratios produced in *B. dorsalis* and *B. cucurbitae*, rectal *Bacillus* species were isolated and tested for their abilities to produce TMP and TTMP. As a result, 7 *Bacillus* strains were isolated from the rectums of *B. dorsalis* (3 strains) and *B. cucurbitae* (4 strains). However, the isolated strains from *B. dorsalis* and *B. cucurbitae* showed no significant phylogenetic difference (Fig. 2D). In vitro *Bacillus* fermentation assays also indicated that all isolated strains produced higher levels of TMP than TTMP, and even the most distantly related strains of *B. dorsalis* and *B. cucurbitae* (BD-3 and BC-3) produced TMP and TTMP in similar ratios (TMP content was much higher than TTMP content) (Fig. 2E). Moreover, we further identified the TMP/TTMP ratios in *B. dorsalis* (or *B. cucurbitae*) that were fed rectal *Bacillus* or microbiota from *B. cucurbitae* (or *B. dorsalis*) to verify whether rectal *Bacillus* or microbiota was responsible for the different TMP/TTMP ratios in *B. dorsalis* and *B. cucurbitae* in vivo. The results showed that feeding one fly (original rectal microbiota was eliminated by feeding streptomycin) with rectal *Bacillus* or microbiota from another fly did not change the TMP/TTMP ratios, TMP/TTMP ratios were still significantly higher in *B. dorsalis* than in *B. cucurbitae* (Fig. 2F, G and Supplementary Fig. S3). These results demonstrated that differences in rectal *Bacillus* are not associated with different TMP/TTMP ratios in *B. dorsalis* and *B. cucurbitae*, although rectal *Bacillus* is responsible for producing TMP and TTMP.

### Different amino acid levels determined the preference of rectal *Bacillus* to produce TMP or TTMP

**Fig. 3:**
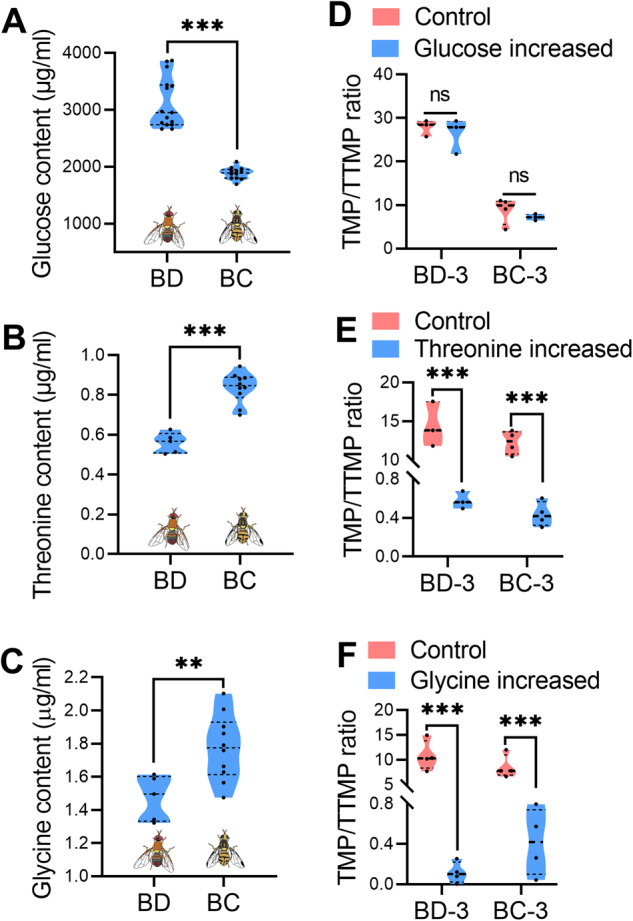
Influence of precursor substance levels on TMP/TTMP ratios produced by *Bacillus*. **A**–**C** Comparison of glucose (*n* ≥ 12 replicates, independent sample *t* test, ***p < 0.001), threonine (*n* = 5 replicates, independent sample *t* test, ***p < 0.001) and glycine (*n* = 5 replicates, independent sample *t* test, ** *p* < 0.01) levels in mature male rectum of *B. dorsalis* and *B. cucurbitae*. **D** TMP/TTMP ratios produced by *Bacillus* comparison when the glucose content in the medium was increased (*n* = 3–4 replicates, independent sample *t* test, “ns” no significance). **E** TMP/TTMP ratios produced by *Bacillus* comparison when the threonine content in the medium was increased (*n* = 3–4 replicates, independent sample *t* test, *** *p* < 0.001). **F** TMP/TTMP ratios *p*roduced by *Bacillus* comparison when the glycine content in the medium was increased (*n* = 4 replicates, independent sample *t* test, *** *p* < 0.001).

In the proposed alkylpyrazine synthesis pathway, *Bacillus* can use glucose and threonine (or glycine) to synthesize TMP and TTMP [16]. Thus, we hypothesized that differences in glucose, threonine and/or glycine levels in the male rectum might affect the TMP/TTMP ratios in *B. dorsalis* and *B. cucurbitae*. Indeed, glucose, threonine and glycine levels in *B. dorsalis* and *B. cucurbitae* male rectum were significantly different. The glucose content was significantly higher in *B. dorsalis*, while the threonine and glycine levels were significantly lower (Fig. 3A–C). To further verify whether glucose, threonine or glycine levels can affect the TMP/TTMP ratios, the TMP/TTMP ratios in the fermentation medium of *Bacillus* were measured after glucose, threonine or glycine content was altered. The results showed that increased glucose content in the medium had no influence on the TMP/TTMP ratios produced by *Bacillus* of *B. dorsalis* and *B. cucurbitae* (Fig. 3D and Supplementary Fig. S4), while increased threonine or glycine content in the medium significantly decreased the TMP/TTMP ratios (Fig. 3E, F, Supplementary Figs. S5 and S6). These results confirm that sex pheromone specificity in *B. dorsalis* and *B. cucurbitae* determined by the different TMP/TTMP ratios may be regulated by different rectal threonine and glycine levels.

### Changed threonine and glycine levels in *B. dorsalis* affect TMP/TTMP ratios and mating

**Fig. 4:**
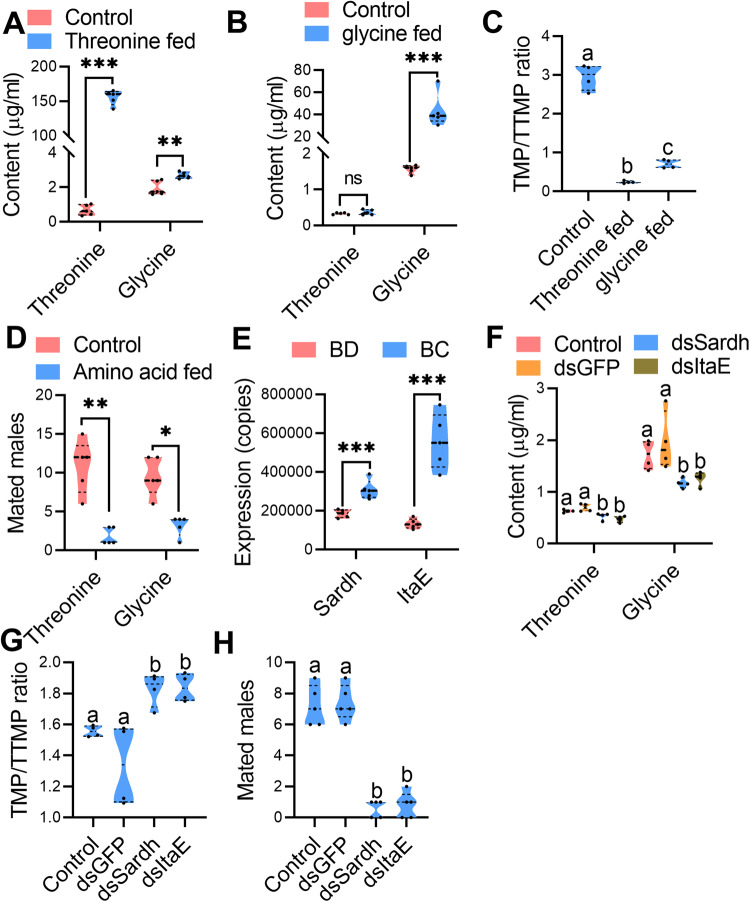
Influence of changes in threonine and glycine levels on the TMP/TTMP ratio and mating of *B. dorsalis*. **A**, **B** Rectal threonine and glycine content comparison between threonine (glycine)-fed *B. dorsalis* and the control (*n* = 5 replicates, independent sample *t* test, ***p < 0.001, **p < 0.01, “ns” no significance). **C** TMP/TTMP ratio comparison between threonine (glycine)-fed *B. dorsalis* and the control (*n* = 4 replicates, different letters indicate significant differences at the 0.05 level, the data were analyzed by ANOVA followed by Tukey’s test). **D** Mating ability comparisons between males fed threonine (glycine) and the control (Threonine group: *n* = 5 replicates, *n* = 5 replicates, paired sample *t* test, *t* = 5.477, **p = 0.0054; Glycine group: *n* = 5 replicates, *n* = 5 replicates, paired sample *t* test, *t* = 4, *p = 0.016). **E** Absolute expression comparison of *Sardh* and ItaE between *B. dorsalis* and *B. cucurbitae* (*n* = 5 replicates, independent sample *t* test, ***p < 0.001). **F** Threonine (*n* = 4 replicates) and glycine (*n* = 4 replicates) levels in the rectum with *Sardh* and *ItaE* knockdown (different letters indicate significant differences at the 0.05 level, the data were analyzed by ANOVA followed by Tukey’s test). **G** TMP/TTMP ratio comparison between *B. dorsalis* with *Sardh* and *ItaE* knockdown and the control (*n* = 4 replicates, different letters above the error bars indicate significant differences at the 0.05 level analyzed by ANOVA followed by Tukey’s test). **H** Mating ability comparisons between males with *Sardh* and *ItaE* knockdown and controls (*n* = 5 replicates, different letters above the error bars indicate significant differences at the 0.05 level analyzed by Kruskal–Wallis test followed by Dunn’s multiple comparisons test).

To further verify the influence of threonine and glycine levels on the TMP/TTMP ratios, threonine- or glycine-rich diets were used to feed *B. dorsalis* to increase rectal threonine or glycine levels, and then TMP/TTMP ratios and mating ability of the males were recorded. As a result, rectal threonine or glycine levels in *B. dorsalis* fed threonine- or glycine-rich diets could be increased significantly (Fig. 4A, B). GC‒MS results indicated that TMP/TTMP ratios in *B. dorsalis* fed threonine- or glycine-rich diets could be completely reversed compared with control males (Fig. 4C and Supplementary Fig. S7). Moreover, the mating ability of the *B. dorsalis* males that were fed threonine- or glycine-rich diets decreased significantly compared with the control males (Fig. 4D).

Studies have indicated that the sarcosine dehydrogenase gene (*Sardh*) and threonine aldolase gene (*ItaE*) in the glycine, serine and threonine metabolism pathway are involved in synthesizing glycine and threonine in insects [17]. Thus, we speculate that higher expression levels of *Sardh* and *ItaE* contribute to the higher levels of glycine and threonine in *B. cucurbitae* than in *B. dorsalis*. To verify this hypothesis, we first quantified and compared the absolute expression of *Sardh* and *ItaE* in the rectum between *B. dorsalis* and *B. cucurbitae*. The results showed that the absolute expression of *Sardh* and *ItaE* in the rectum of *B. cucurbitae* was indeed significantly higher than that in *B. dorsalis* (Fig. 4E). Next, we knocked down the expression of *Sardh* and *ItaE* in *B. dorsalis* (Supplementary Fig. S8) to decrease rectal threonine or glycine levels in male *B. dorsalis*, and then TMP/TTMP ratios and mating ability of the males were recorded. The results indicated that *B. darsalis* male rectal glycine or threonine levels could be decreased significantly when *Sardh* or *ItaE* was knocked down (Fig. 4F). Accordingly, TMP/TTMP ratios increased significantly in males with *Sardh* or *ItaE* knockdown (though both TMP and TTMP levels were decreased) (Fig. 4G and Supplementary Fig. S9). The mating competition results showed that the mating ability of males with *Sardh* or *ItaE* knockdown decreased significantly (Fig. 4H).

Together, these results confirmed our original hypothesis that TMP/TTMP ratios and mating ability of *B. dorsalis* males are associated with rectal threonine and glycine levels, and *Sardh* and *ItaE* play important roles in converting threonine and glycine in the rectum of *B. dorsalis*.

### Changed threonine and glycine levels in *B. cucurbitae* affect TMP/TTMP ratios and mating

**Fig. 5:**
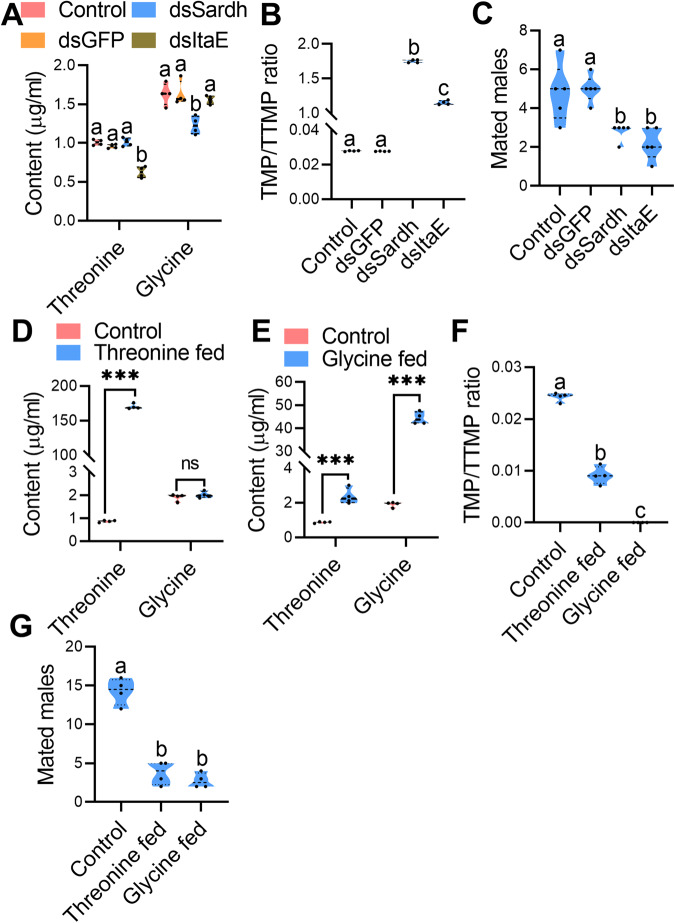
Influence of threonine and glycine levels on TMP/TTMP ratios and mating in *B. cucurbitae*. **A** Threonine (*n* = 4 replicates) and glycine (*n* = 4 replicates) levels in the rectum with *Sardh* and *ItaE* knockdown (different letters indicate significant differences at the 0.05 level, the data were analyzed by ANOVA followed by Tukey’s test). **B** TMP/TTMP ratio comparison between *B. cucurbitae* with *Sardh* and *ItaE* knockdown and the control. **C** Mating ability comparisons between males with *Sardh* and *ItaE* knockdown and controls (*n* = 5 replicates, different letters above the error bars indicate significant differences at the 0.05 level analyzed by Kruskal–Wallis test followed by Dunn’s multiple comparisons test). **D**, **E** Rectal threonine and glycine content comparison between threonine (glycine)-fed *B. cucurbitae* and the control (*n* = 4 replicates, independent sample *t* test, ***p < 0.001, **p < 0.01, “ns” no significance). **F** TMP/TTMP ratio comparison between threonine (glycine)-fed *B. cucurbitae* and the control (*n* = 4 replicates, different letters indicate significant differences at the 0.05 level, the data were analyzed by ANOVA followed by Tukey’s test). **G** Mating ability comparisons between males fed threonine (glycine) and the control (*n* = 5 replicates, paired sample *t* test, **p < 0.01).

For *B. cucurbitae*, we also changed the rectal threonine (glycine) content to verify its influence on TMP/TTMP ratios. First, we knocked down *Sardh* and *ItaE* with RNAi (Supplementary Fig. S10) to decrease rectal threonine or glycine levels in male *B. cucurbitae*, and then TMP/TTMP ratios and mating ability of the males were recorded. The results showed that *B. cucurbitae* male rectal glycine or threonine levels could be decreased significantly when *Sardh* or *ItaE* was knocked down (Fig. 5A). Accordingly, TMP/TTMP ratios were increased significantly in males with *Sardh* or *ItaE* knockdown (Fig. 5B and Supplementary Fig. S11). Mating competition results also showed that the mating ability of the males with *Sardh* or *ItaE* knocked down decreased significantly (Fig. 5C).

In contrast to decreasing rectal threonine or glycine levels by knocking down *Sardh* or *ItaE*, threonine- or glycine-rich diets were used to feed *B. cucurbitae* to increase rectal threonine or glycine levels, and then TMP/TTMP ratios and mating ability of the males were recorded. As a result, rectal threonine or glycine levels in *B. cucurbitae* fed threonine- or glycine-rich diets could be increased significantly (Fig. 5D, E). Accordingly, TMP/TTMP ratios decreased significantly in males fed threonine- or glycine-rich diets (almost no TMP was detected in threonine- or glycine-fed males) (Fig. 5F and Supplementary Fig. S12). Meanwhile, the mating competition results showed that the mating ability of the males fed threonine or glycine decreased significantly (Fig. 5G).

Together, our results demonstrated that TMP/TTMP ratios and mating ability of *B. cucurbitae* males are also associated with rectal threonine and glycine levels, and *Sardh* and *ItaE* play an important role in converting threonine and glycine in the rectum of *B. cucurbitae*.

## Discussion

**Fig. 6:**
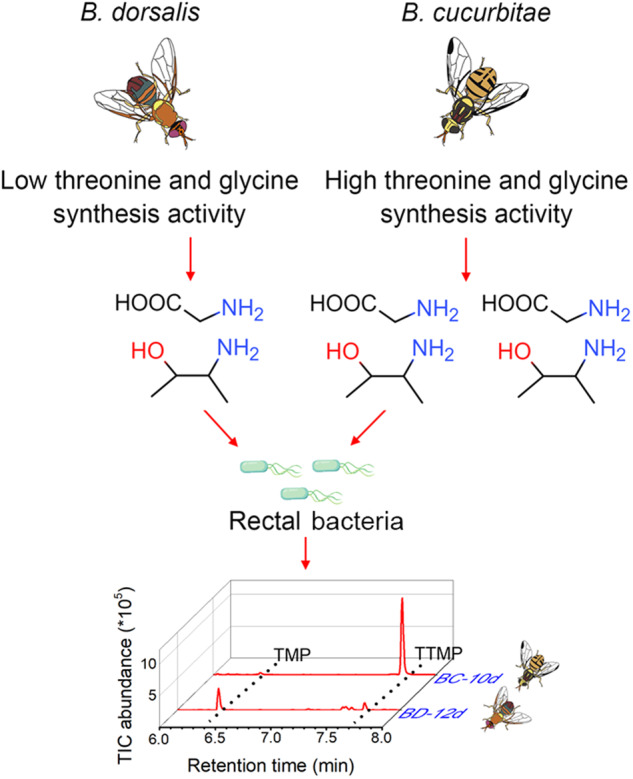
Schematic illustrating the sex pheromone specificity generation hypothesis between *B. dorsalis* and *B. cucurbita*e. In *B. dorsalis*, low *Sardh* and *ItaE* gene expression results in lower threonine and glycine synthesis activity and thus leads to a higher TMP/TTMP ratio produced by rectal bacteria. In contrast, higher *Sardh* and *ItaE* gene expression results in higher threonine and glycine synthesis activity and thus leads to a lower TMP/TTMP ratio produced by rectal bacteria.

In most animals, sexual selection, which coevolved from a female preference for certain male traits, led to rapid and dramatic evolutionary divergence [18–20] and even contributed to speciation [21]. Using 16S rRNA gene amplicon sequencing combined with bacterial fermentation, we investigated how the male sex pheromones of *B. dorsalis* and *B. cucurbitae* are produced. By linking chemical variations with the rectal amino acid levels and the associated pathway, we provided evidence that rectal amino acids determined the differentiation of sex pheromones between two closely related tephritid flies (Fig. 6). The characterization of sex pheromones and the divergence of their origin among the studied tephritid flies provide several new insights into the evolution of sex pheromone production and the role of sex pheromones in speciation.

In recent decades, a large number of studies have indicated that sex pheromone biosynthesis is related to metabolism-related genes and endocrine regulation in insects [22–24]. It appears that insects have evolved to produce one or a few tissue-specific auxiliary or modified enzymes that transform the products of “normal” metabolism into pheromone compounds of high stereochemical and quantitative specificity in pheromone glands. In this study, we have proposed a new way to create pheromone diversity in tephritid flies. Specifically, differences in amino acid synthesis in the rectum can lead to differences in the proportion of sex pheromones produced by rectal bacteria in closely related species. Although some other insect pheromones are also hypothesized to be of microbial origin, direct evidence for the mechanism underlying microbial production remains scarce. We have demonstrated here that *Bacillus* from the rectums of *B. dorsalis* and *B. cucurbitae* can produce sex pheromones (TMP and TTMP) in vitro. Studies have revealed that many insects use pyrazines as pheromones [25–28]. However, no studies have indicated that pyrazines can be synthesized by insects until now. Thus, we believe that bacteria-derived pyrazines may be widespread in insects.

The species specificity of sex pheromones is conferred by the combination of volatile compounds at different ratios. Changes in sex pheromone composition can lead to reproductive isolation between closely related species [8, 29]. In general, changes in volatile compound levels among related species are related to genetic relationships [30], geographical locations [31] and environmental factors [32]. Genetic relatedness can even determine the opposite ratios of sex pheromone components [33]. For example, in moths, enzymes (mainly desaturase and fatty acyl reductase) have been found to account for pheromone differences between closely related species [34, 35]. Our findings revealed that male-borne sex pheromones are conserved between *B. dorsalis* and *B. cucurbitae*, and that sex pheromones can be produced by *Bacillus* in the rectum of *B. dorsalis* and *B. cucurbitae*. Differences in the rectal threonine and glycine levels were responsible for differences in sex pheromone ratios between *B. dorsalis* and *B. cucurbitae*. Moreover, different expression efficiencies of the enzyme in the threonine- and glycine-synthesizing pathways determine different rectal threonine and glycine levels, which in turn affect the sex pheromone ratios. In tephritid flies, protein feeding after emergence can significantly affect sexual performance [36, 37]. Therefore, differential expression patterns of enzymes that hydrolyze proteins into threonine and glycine may also affect the threonine and glycine levels in *B. dorsalis* and *B. cucurbitae* and thus affect the TMP/TTMP ratios.

Modifications of sex pheromones are thought to represent a rapid means for altering mate recognition abilities during the evolution of new species [38]. Our results provide insights into how interspecific sexual barriers arise. Although the two male-specific pyrazines are both produced by *B. dorsalis* and *B. cucurbitae* males, the ratios of these compounds are dramatically different. This marked difference in the chemical ratios is unexpected given that the adult flies are bred on the same food in nature and were bred on the same food in our study. The speciation history of *Bactrocera* is characterized by sudden and profound changes in host plant (larval stage) use [39]. Changes in host plant use are important factors in speciation [40]. Moreover, host adaptation can lead to reproductive isolation characterized by reproductive character displacement, including in sexual signals [41]. Host plant-related chemical differences in *Bactrocera* may even be affected by diet-related precursors. At the adult stage, *Bactrocera* use almost the same diet (mainly nectar). Therefore, *B. dorsalis* and *B. cucurbitae* have the same pheromone profiles, despite being phylogenetically separate. Given that the ratios of the pheromones were dramatically different, we argue that changes in the sex pheromone profiles of *B. dorsalis* and *B. cucurbitae* are associated not only with the host plant but also with genetic divergence. Our results may indicate a mode of evolution whereby rapid changes in chemical composition occur during speciation events, and greater divergence gradually occurs subsequently.

TMP/TTMP ratios differ in *B. cucurbitae* and *B. dorsalis*, many issues still need to be resolved. In addition to different threonine and glycine levels, we found that rectal glucose differs significantly even if both species are maintained on the same diet. In insects, both diet and sugar metabolism can significantly affect glucose levels [42–44]. Given that both species were fed the same diet, we speculate that differences in glucose metabolism and transport efficiency might be the reason for different rectal glucose levels. Moreover, we found that TMP/TTMP ratios in *B. dorsalis* fluctuate widely between the results in this study and our previous study [13]. Given that amino acid levels in the diet can significantly affect TMP/TTMP ratios (Fig. 4C and Fig. 5F), it is possible that this could be responsible for such differences in our previous study [13]. Amino acids in the diet were derived from yeast extracts purchased from a local market, and different batches could have caused the amino acid level differences in different studies. Although the ratios fluctuated in different studies, the ratios between the two flies fed with the same diet was reversed both in wild and lab populations. Moreover, in females, a similar level of attraction to TMP or TTMP alone compared to that with a mixture of TMP and TTMP was detected using a Y-shaped olfactometer [13]. However, we found that a change in TMP/TTMP ratios in males could result in significant mating efficiency in both flies (Fig. 4 and Fig. 5). In Tephritidae, successful mating between males and females can only be achieved with sex pheromones, auditory signals and visual signals together [9]. Therefore, the actual attraction to different ratios of sex pheromones in females needs to be tested in the presence of auditory and visual signals. Moreover, our results showed that the TMP/TTMP ratio is decreased (or increased) in males of *B. dorsalis* (or *B. cucurbitae*) upon TMP or TTMP addition to the abdomen, which significantly decreased attraction to females and thus reduced male mating ability (Fig. 1H, I).

## Materials and methods

### Insect preparation

The *B. dorsalis* and *B. cucurbitae* strains were reared under laboratory conditions (27 ± 1 °C, 12:12 h light:dark cycle, 70–80% RH). Guava and cocozelle were used to feed the larvae of *B. dorsalis* and *B. cucurbitae*, respectively. Adults were fed a diet consisting of a 1:1 ratio of yeast extract (protein food):sugar in a 35 cm × 35 cm × 35 cm wooden cage. For antibiotic treatment, newly emergent male flies were continuously fed streptomycin (3 mg/ml, diluted in sterile water).

To measure TMP/TTMP ratios in wild flies, *B. dorsalis* and *B. cucurbitae* males were collected with the male specific lure-methyl eugenol and raspberry ketones, respectively.

To verify if sex pheromone specificity between *B. dorsalis* and *B. cucurbitae* is conferred by different TMP/TTMP ratios, mature males added with TMP or TTMP solutions were prepared. Briefly, 1 μl TTMP solution or TMP solution (2 μg/μl, diluted in ethanol) was added on the abdomen of the mature male of *B. dorsalis* or *B. cucurbitae* to decrease and increase TMP/TTMP ratios in *B. dorsalis* and *B. cucurbitae*, respectively. Males added with 1 μl ethanol were prepared as control. Then attraction and mating abilities of the males were tested with the mating competition recording method.

### Attraction of mature females to rectal extracts

The rectums of 60 males at different developmental stages (*B. dorsalis*: 0, 3, 6 and 12 days old; *B. cucurbitae*: 0, 2, 6 and 10 days old) were dissected and extracted with 500 µl ethanol for 24 h. The attraction effects of the prepared extracts for mature females were tested in a Y-shaped olfactometer [13]. Briefly, 500 µl of extract in a 1.5 ml vial was placed in an odor-controlled glass chamber. Then, 12-day-old (for *B. dorsalis*) or 10-day-old (for *B. cucurbitae*) unmated females were individually released at the base of the olfactometer and allowed 5 min to show a selective response. Ethanol was used as control. Other procedures were performed as described in a previous study [13]. More than 40 females were selected for testing, and each female was used only once for each odor.

### Rectal volatile compound identification

The volatile compounds present in the extracts prepared as described above were identified by GC–MS (Agilent 7890B Series GC system coupled to a quadrupole-type-mass-selective detector (Agilent 5977B; transfer line temperature: 230 °C, source temperature: 230 °C, ionization potential: 70 eV)). The volatile compounds were chromatographed on an HP-5 MS column (30 m length, 0.25 mm internal diameter, 0.25 μm film thickness). Helium at a constant pressure of 110.9 kPa was used as a carrier gas. One microliter of extract was automatically injected into the injector port (240 °C). The column temperature was maintained at 50 °C for 1 min, increased to 150 °C at 10 °C/min, increased to 250 °C at 25 °C/min and maintained at 250 °C for 10 min. The mass spectra of the chemicals were compared with those listed in the NIST mass spectral library. Then, the identification of TMP and TTMP was confirmed by comparing their retention times and mass spectra with those of authentic standards.

To identify the volatile compounds in the bacterial fermentation broth, a 100-μm polydimethylsiloxane (PDMS) SPME fiber (Supelco) was used to capture the volatile compounds for 30 min at room temperature. Then, the compounds were analyzed by GC–MS according to the methods described above.

### Mating competition

Mating competition assays were performed in a 35 cm × 35 cm × 35 cm wooden cage according to our previous study [45]. Briefly, male pronota were colored and placed in one cage, and then 30 mature unmated females were placed in the cage at 8:00 p.m. Mating behavior was observed for 2 h, and the number of mated males was recorded and compared. In each assay, 30 males were prepared for each group.

### Determination of rectal bacterial community composition by 16S rRNA gene amplicon sequencing

To analyze bacterial diversity in the male rectum, the rectums of 5 mature males (for B. dorsali, 12-day-old male; for *B. cucurbitae*, 10-day-old male) were collected (five replicate samples were prepared). Then, bacterial DNA was extracted from the rectum samples using the Bacterial Genomic DNA Extraction Kit (Tiangen, Beijing, China) according to the manufacturer’s protocol. To analyze the bacterial diversity in the male rectum, the 16S rRNA V3-V4 region was amplified by PCR. Then, the amplicons were purified and sequenced (2 × 250) on a HiSeq 2500 System (Illumina). The software Mothur was used to cluster tags of more than 97% identity into OTUs, and then the abundances of the OTUs were calculated. The taxonomic classification of OTUs was based on the annotation result of contained tags according to the mode principle; that is, the taxonomic rank that contained more than 66% of tags was considered the taxonomic rank of a specific OTU. Alpha diversity represents bacterial diversity in a single sample and was evaluated by the Shannon index. The Shannon index is a diversity index that considers OTU abundance and OTU evenness.

### Isolation and identification of *Bacillus* in the rectum

For isolation of bacteria from the male rectum, the rectums of 5 mature males were dissected and collected in a sterile centrifuge tube, to which 1 ml of sterile water was added. The rectums were then ground with sterile grinding pestles and shaken for 20 min. A 200 µl volume of diluted liquid was then used to coat an LB plate, and the plate was cultured for 1 day. Pure bacterial colonies were selected by subculturing on LB medium and stored in a 25% glycerol solution at −80 °C. The Bacterial Genomic DNA Extraction Kit (Tiangen, Beijing, China) was used to extract bacterial DNA according to the manufacturer’s instructions. Universal primers (F: 5′-AGAGTTTCATCCTGGCTCAG-3′ and R: 5′-TACGGTTAXXTTGTTACGACTT-3′) were used to amplify 16S rRNA. The PCR products were confirmed by electrophoresis on a 0.8% agarose gel, and the target PCR product was sequenced. The 16S rRNA sequence was BLAST searched against the NCBI NR database (https://blast.ncbi.nlm.nih.gov/Blast.cgi). The isolates identified as *Bacillus* were used for subsequent assays. Moreover, phylogenetic analysis was performed for the strains isolated from *B. dorsalis* and *B. cucurbitae* with MEGA 7.0.

### Cultivation experiments

Cultivation experiments were conducted in 250-ml shake flasks containing 50 ml of LB medium supplemented with membrane filtration-sterilized threonine (42 mM), glucose (56 mM), and (NH4)_2_HPO_4_ (23 mM) to study the production of TMP and TTMP by *Bacillus* isolated from the rectums of *B. dorsalis* and *B. cucurbitae*. To test the effect of glucose, threonine and glycine content on the TMP/TTMP ratio produced by *Bacillus*, LB medium supplemented with more threonine (70 mM), glycine (70 mM) or glucose (84 mM) was prepared for TMP and TTMP fermentation. *Bacillus* was inoculated in medium at a concentration of 1% and cultured aerobically at 37 °C with shaking at 200 rpm for 2 days. Experiments were conducted in triplicate.

### TMP/TTMP ratio identification in *B. dorsalis* (*B. cucurbitae*) after feeding on microbiota or *Bacillus* from *B. cucurbitae* (*B. dorsalis*)

To show whether the difference in rectal microbiota or *Bacillus* is associated with different TMP/TTMP ratios in *B. dorsalis* and *B. cucurbitae*, newly emerged males of *B. dorsalis* and *B. cucurbitae* were fed streptomycin (3 mg/ml diluted in sterile water), which was effective in eliminating rectal microbiota [13]. Ten days after streptomycin treatment, the rectal microbiota-eliminated flies were fed microbiota or *Bacillus* isolated from the other flies (*B. dorsalis* (*B. cucurbitae*) fed microbiota or *Bacillus* isolated from *B. cucurbitae* (*B. dorsalis*)). Then, the rectums of the mature flies (2 days after *Bacillus* feeding) were collected to extract sex pheromones. The TMP/TTMP ratio in the rectum was identified with GC‒MS as described above. To collect rectal microbiota, 60 rectums of *B. dorsalis* or *B. cucurbitae* were dissected and grinded. Then 1 ml sterile water was used to wash out the microbiota. The collected microbiota was used to feed the flies for 2 days.

### Measurement of glucose, threonine and glycine levels in the rectum of *B. dorsalis* and *B. cucurbitae* males

Glucose, threonine and glycine levels in the rectum of mature *B. dorsalis* and *B. cucurbitae* males were measured. The glucose content in the rectum was measured with a glucometer (ONETOUCH, Verio Flex). Briefly, the rectums of 12 mature *B. dorsalis* and *B. cucurbitae* males was collected and placed in a 1.5-ml microcentrifuge tube containing 10 μl of sterile Milli-Q water. Then, the samples were ground with a grinding machine. The samples were centrifuged for 15 min at 12000 rpm. Then, the supernatants were collected and analyzed with a glucometer. For threonine and glycine identification, sample preparation for free amino acid analysis was performed as described by [46]. Briefly, the rectums of 15 mature males were collected and placed in a 1.5-ml microcentrifuge tube containing 500 μl of sulfosalicylic acid solution (5%, diluted in water). Then, the samples were ground with a grinding machine. The samples were centrifuged for 15 min at 12000 rpm. Then, the supernatants were collected in another centrifuge tube, and 1 ml of sulfosalicylic acid solution was added. Then, the threonine and glycine levels in the samples were quantified with an amino acid analyzer (Hitachi L-8900, Japan) according to the standard method.

### TMP/TTMP ratio identification in *B. dorsalis* and *B. cucurbitae* with threonine and glycine changed

To increase threonine or glycine content in males of *B. dorsalis* and *B. cucurbitae*, newly emerged males were fed a normal diet and threonine or glycine solution (diluted in sterile water, 10 μg/ml) as a water source. After the males matured, the TMP/TTMP ratio in the rectum was identified with GC‒MS as described above. In addition, normally reared males were prepared as controls.

### Absolute expression of *Sardh* and *ItaE* verification in *B. dorsalis* and *B. cucurbitae*

Total RNA was extracted from the rectum of mature *B. dorsalis* and *B. cucurbitae* males. Then, cDNA was synthesized with a One-Step gDNA Removal and cDNA Synthesis SuperMix Kit (TransGen Biotech, Beijing, China) using the extracted RNA. Specific primers (Supplementary Table S1) for the *Sardh* and *ItaE* genes of *B. dorsalis* and *B. cucurbitae* were designed to quantify the absolute expression of *Sardh* and *ItaE* in the mature male rectum. The cDNA was used for amplification with the primers. The amplified fragment was then cloned into the pMD 18-T vector, which was then transferred into *E. coli* DH5α to reproduce. The reproduced vector was then extracted with a plasmid extraction kit and diluted in a series of 10-fold dilutions to obtain 5 different plasmid concentrations (measured by a Nanodrop spectrophotometer). A standard curve for qPCR was then generated by amplifying the 16S rRNA of the plasmid. The absolute expression of *Sardh* and *ItaE* in *B. dorsalis* and *B. cucurbitae* was then determined by referring to the standard curve with the CT values.

### RNA interference

Double-stranded RNA (dsRNA) primers (Supplementary Table S1) tailed with the T7 promoter sequence were designed using the CDSs of *Sardh* and *ItaE* of *B. dorsalis* and *B. cucurbitae* (GenBank accession number: XM_029043528.1, XM_011193687.2, XM_011208969.4 and XM_011205849.4) as templates. A MEGAscript RNAi Kit (Thermo Fisher Scientific, United States) was used to synthesize and purify dsRNA according to the manufacturer’s instructions. The GFP gene (GenBank accession number: AHE38523) was used as the RNAi negative control. To knock down the target gene in males, 0.5 μL (500 ng/μl) dsRNA was injected into the abdomen of mature males. Normal flies and flies injected with dsGFP were prepared as negative controls. After 24 h, the knockdown efficiency of the genes was checked with qRT‒PCR following the method used for validating the expression of the gene. Then, the threonine and glycine levels, sex pheromones and mating ability were measured and tested.

### Relative expression validation of the identified genes

qRT‒PCR analysis was used to validate gene expression in *Sardh*-silenced males of *B. cucurbitae*. Total RNA was extracted. Then, cDNA was synthesized with a One-Step gDNA Removal and cDNA Synthesis SuperMix Kit (TransGen Biotech, Beijing, China) using the extracted RNA. Then, a PerfectStarTM Green qPCR SuperMix Kit (TransGen Biotech, Beijing, China) was used to perform quantitative real-time PCR to compare the gene expression levels. Gene-specific primers (Supplementary Table S1) were designed on NCBI with primer blast. The *RPL60* gene was used as a reference gene [47]. The PCR procedure was set according to the manufacturers’ instructions. Five biological replicates were performed.

## Supplementary information

supplementary figures and tables

Dataset S1

Dataset S2

## Data Availability

All data needed to evaluate the conclusions in the paper are present in the paper and/or the Supplementary Materials. The 16S rRNA amplicon sequencing data have been uploaded to the National Genomics Data Center with accession numbers CRA006849 and CRA006854. 16S rRNA sequences of the isolated *Bacillus* were unloaded to the National Genomics Data Center with accession numbers ON819613, ON819614, ON819615, ON819616, ON819617, ON819618 and ON819613.

## References

[1] Karlson P, Luscher M (1959). Pheromones: a new term for a class of biologically active substances. Nature..

[2] De Pasqual C, Groot AT, Mappes J, Burd E (2021). Evolutionary importance of intraspecific variation in sex pheromones. Trends Ecol Evol.

[3] Linn CE, Roelofs WL (1985). Response specificity of male pink bollworm moths to different blends and dosages of sex pheromone. J Chem Ecol.

[4] Jensen K, Shearman M, Rapkin J, Carey MR, House CM, Hunt J (2017). Change in sex pheromone expression by nutritional shift in male cockroaches. Behav Ecol.

[5] Balmer AJ, Brakefield PM, Brattstrom O, van Bergen E (2018). Developmental plasticity for male secondary sexual traits in a group of polyphenic tropical butterflies. Oikos..

[6] Groot AT, Classen A, Staudacher H, Schal C, Heckel DG (2010). Phenotypic plasticity in sexual communication signal of a noctuid moth. J Evol Biol.

[7] Schultzhaus JN, Bennett CJ, Iftikhar H, Yew JY, Mallett J, Carney GE (2018). High fat diet alters *Drosophila melanogaster* sexual behavior and traits: decreased attractiveness and changes in pheromone profiles. Sci Rep.

[8] Roelofs WL, Liu WT, Hao GX, Jiao HM, Rooney AP, Linn CE (2002). Evolution of moth sex pheromones via ancestral genes. Proc Natl Acad Sci USA.

[9] Benelli G, Daane KM, Canale A, Niu C-Y, Messing RH, Vargas RI (2014). Sexual communication and related behaviours in Tephritidae: current knowledge and potential applications for Integrated Pest Management. J Pest Sci.

[10] Suzuki Y, Koyama J (1980). Temporal aspects of mating behavior of the melon fly, *Dacus cucurbitae* COQUILLETT (Diptera : Tephritidae) : a comparison between laboratory and wild strains. Appl Entomol Zool.

[11] Baker R, Herbert RH, Lomer RA (1982). Chemical components of the rectal gland secretions of male *Dacus cucurbitae*, the melon fly. Experientia..

[12] Perkins MV, Fletcher MT, Kitching W, Drew RA, Moore CJ (1990). Chemical studies of rectal gland secretions of some species of *Bactrocera dorsalis* complex of fruit flies (diptera: Tephritidae). J Chem Ecol.

[13] Ren L, Ma Y, Xie M, Lu Y, Cheng D (2021). Rectal bacteria produce sex pheromones in the male oriental fruit fly. Curr Biol.

[14] Klosowski G, Mikulski D, Pielech-Przybylska K (2021). Pyrazines biosynthesis by *Bacillus* strains isolated from natto fermented soybean. Biomolecules..

[15] Xiao ZJ, Xie NZ, Liu PH, Hua DL, Xu P (2006). Tetramethylpyrazine production from glucose by a newly isolated *Bacillus* mutant. Appl Microbiol Biot.

[16] Zhang L, Cao Y, Tong J, Xu Y (2019). An alkylpyrazine synthesis mechanism involving L-threonine-3-dehydrogenase describes the production of 2,5-dimethylpyrazine and 2,3,5-trimethylpyrazine by *Bacillus subtilis*. Appl Environ Micro.

[17] Frisell WR, Mackenzie CG (1962). Separation and purification of sarcosine dehydrogenase and dimethylglycine dehydrogenase. J Biol Chem.

[18] Lande R (1981). Models of speciation by sexual selection on polygenic traits. Proc Natl Acad Sci USA.

[19] Ritchie MG (2007). Sexual selection and speciation. Annu Rev Ecol Evol S..

[20] Servedio MR, Boughman JW (2017). The role of sexual selection in local adaptation and speciation. Annu Rev Ecol Evol S..

[21] Mayr E. Systematics and the origin of species. New York: Columbia University Press; 1942.

[22] Jurenka R (2017). Regulation of pheromone biosynthesis in moths. Curr Opin Insect Sci.

[23] Raina AK (1993). Neuroendocrine control of sex pheromone biosynthesis in Lepidoptera. Annu Rev Entomol.

[24] Tillman JA, Seybold SJ, Jurenka RA, Blomquist GJ (1999). Insect pheromones–an overview of biosynthesis and endocrine regulation. Insect Biochem Molec.

[25] Tentschert J, Bestmann HJ, Holldobler B, Heinze J (2000). 2,3-dimethyl-5-(2-methylpropyl)pyrazin, a trail pheromone component of *Eutetramorium mocquerysi* Emery (1899) (Hymenoptera : Formicidae). Naturwissenschaften.

[26] Meer RKV, Preston CA, Choi M-Y (2010). Isolation of a pyrazine alarm pheromone component from the fire ant, *Solenopsis invicta*. J Chem Ecol.

[27] Robledo N, Vega M, Escalante J, Arzuffi R (2014). A new component of the male papaya fruit fly (Diptera: Tephritidae) sex pheromone. Fla Entomol.

[28] Mori K, Yang CY (2017). Pheromone synthesis. Part 261: synthesis of four pyrazines produced by females of the Korean apricot wasp, *Eurytoma maslovskii*. Tetrahedron.

[29] Allison JD, Card RT (2016). Pheromone communication in moths.

[30] van Wilgenburg E, Symonds MRE, Elgar MA (2011). Evolution of cuticular hydrocarbon diversity in ants. J Evol Biol.

[31] Akino T, Yamamura K, Wakamura S, Yamaoka R (2004). Direct behavioral evidence for hydrocarbons as nestmate recognition cues in *Formica japonica* (Hymenoptera : Formicidae). Appl Entomol Zool.

[32] Leonhardt SD, Rasmussen C, Schmitt T (2013). Genes versus environment: geography and phylogenetic relationships shape the chemical profiles of stingless bees on a global scale. P R Soc B Biol Sci.

[33] Li R-T, Ning C, Huang L-Q, Dong J-F, Li X, Wang C-Z (2017). Expressional divergences of two desaturase genes determine the opposite ratios of two sex pheromone components in *Helicoverpa armigera* and *Helicoverpa assulta*. Insect Biochem Molec.

[34] Knipple DC, Rosenfield CL, Nielsen R, You KM, Jeong SE (2002). Evolution of the integral membrane desaturase gene family in moths and flies. Genetics..

[35] Lassance J-M, Lienard MA, Antony B, Qian S, Fujii T, Tabata J (2013). Functional consequences of sequence variation in the pheromone biosynthetic gene pgFAR for *Ostrinia* moths. Proc Natl Acad Sci USA.

[36] Perez-Staples D, Prabhu V, Taylor PW (2007). Post-teneral protein feeding enhances sexual performance of Queensland fruit flies. Physiol Entomol.

[37] Shelly TE (2017). Yeast hydrolysate deprivation and the mating success of male melon flies (Diptera: Tephritidae). Fla Entomol.

[38] Smadja C, Butlin RK (2009). On the scent of speciation: the chemosensory system and its role in premating isolation. Heredity..

[39] Clarke AR, Armstrong KF, Carmichael AE, Milne JR, Raghu S, Roderick GK (2005). Invasive phytophagous pests arising through a recent tropical evolutionary radiation: The *Bactrocera dorsalis* complex of fruit flies. Annu Rev Entomol.

[40] Dres M, Mallet J (2002). Host races in plant-feeding insects and their importance in sympatric speciation. Philos T R Soc B..

[41] Rundle HD, Chenoweth SF, Doughty P, Blows MW (2005). Divergent selection and the evolution of signal traits and mating preferences. Plos Biol.

[42] Kellett GL, Brot-Laroche E, Mace OJ, Leturque A (2008). Sugar absorption in the intestine: the role of GLUT2. Annu Rev Nutr.

[43] Kikuta S, Kikawada T, Hagiwara-Komoda Y, Nakashima N, Noda H (2010). Sugar transporter genes of the brown planthopper, *Nilaparvata lugens*: a facilitated glucose/fructose transporter. Insect Biochem Molec.

[44] MacDonald AJ, Yang YHC, Cruz AM, Beall C, Ellacott KLJ (2021). Brain-body control of glucose homeostasis-insights from model organisms. Front Endocrinol.

[45] Gui S, Yuval B, Engl T, Lu Y, Cheng D (2023). Protein feeding mediates sex pheromone biosynthesis in an insect. Elife..

[46] Shahzad R, Khan AL, Waqas M, Ullah I, Bilal S, Kim Y-H (2019). Metabolic and proteomic alteration in phytohormone-producing endophytic *Bacillus amyloliquefaciens* RWL-1 during methanol utilization. Metabolomics..

[47] Zhang Y, Gong Z, Li L, Niu L, Fu Y (2018). Evaluation of endogenous reference genes in *Bactrocera cucurbitae* by qPCR under different conditions. Plos One.

