# Supplementary material for: Differences in rectal amino acid levels determine bacteria-originated sex pheromone specificity in two closely related flies
Source: ISME J. 2023 Aug 7;17(10):1741–50. doi: 10.1038/s41396-023-01488-9 (PMC10504272; doi:10.1038/s41396-023-01488-9)
Supplement: Supplementary file 1 — supplementary figures and tables [file 41396_2023_1488_MOESM1_ESM.docx]

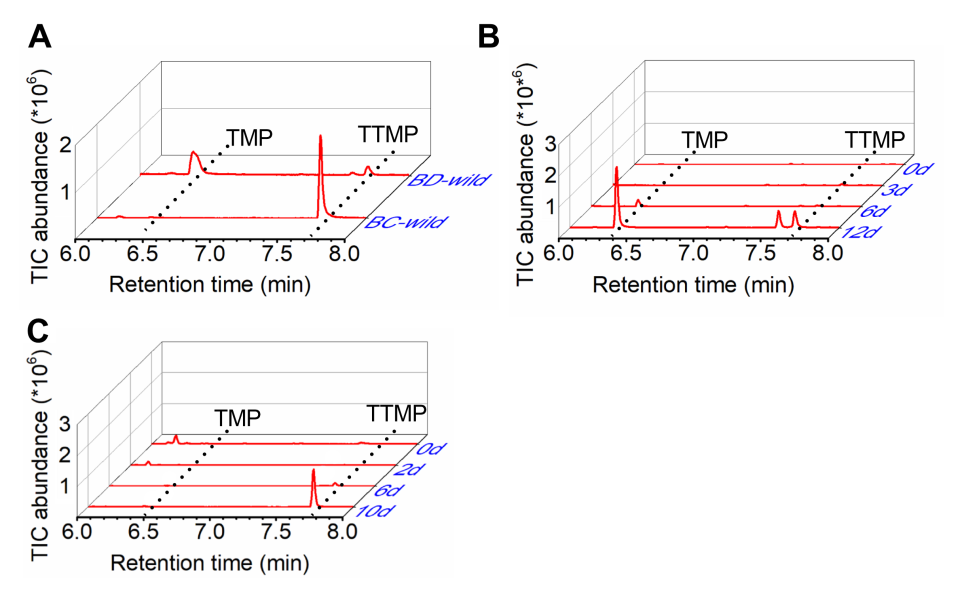


**Fig. S1 BD and BC rectum volatile identification.** (A) Flame ionization detection (FID) traces from GC–MS analysis of male rectal extracts from wild polulation of BD and BC. (B) Flame ionization detection (FID) traces from GC–MS analysis of BD male rectal extracts at different developmental time. (C) Flame ionization detection (FID) traces from GC–MS analysis of BC male rectal extracts at different developmental time. Traces for the flies expressing TMP and TTMP are shown with dotted line.

**
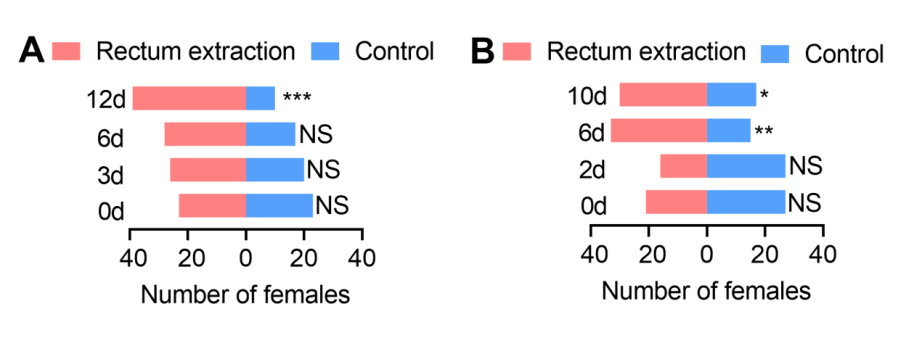
**

**Fig. S2 Attraction effect of BD and BC rectum extraction to mature virgin females.** A chi-square test was used to analyze the selective preference of females in the Y-shaped olfactometer. Stars indicate significant differences relative to the control group. *** *P* < 0.001; ** *P* < 0.01; * *P* < 0.01; NS: no significance.


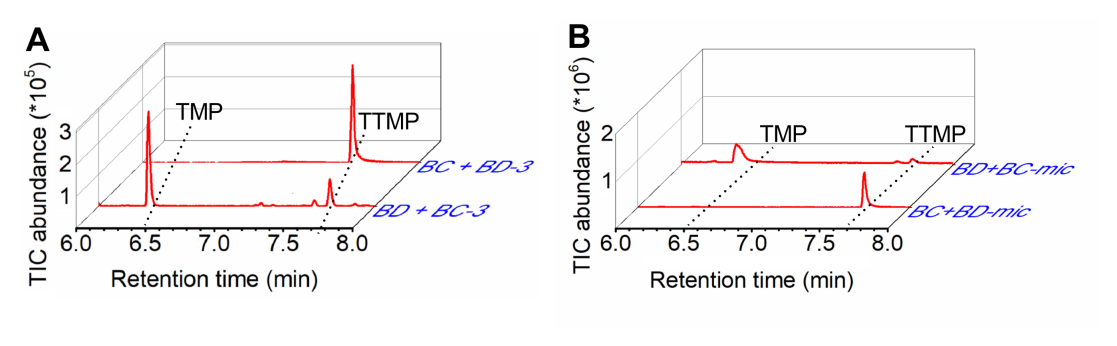


**Fig. S3 GC-MS ion chromatograms of TMP and TTMP identified in the mature male rectum extracts of *B. dorsalis* (BD) (*B. cucurbitae* (BC)) fed with *Bacillus* (A) or microbiota (B) collected from *B. cucurbitae* (*B. dorsalis*).** Traces for TMP and TTMP are shown with dotted line.


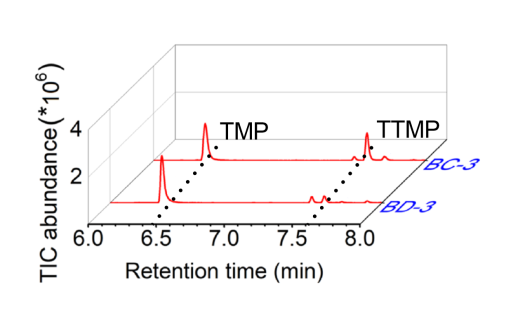


**Fig. S4 TMP and TTMP produced by *Bacillus* when the glucose content in medium was increased.** Traces for TMP and TTMP are shown with dotted line.


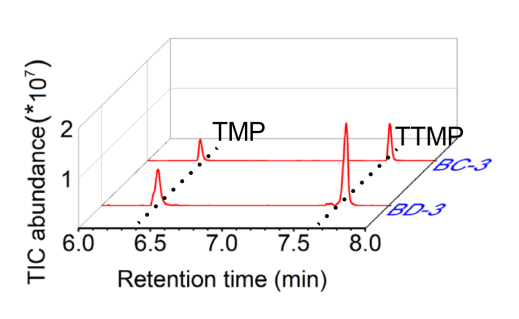


**Fig. S5 TMP and TTMP produced by *Bacillus* when the threonine content in medium was increased.** Traces for TMP and TTMP are shown with dotted line.


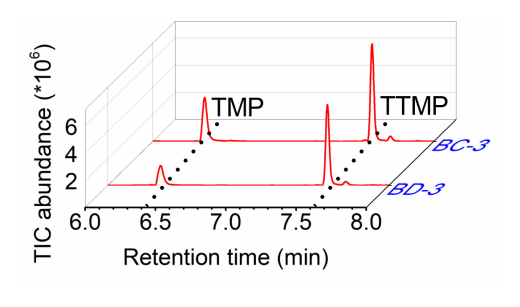


**Fig. S6 TMP and TTMP produced by *Bacillus* when the glycine content in medium was increased.** Traces for TMP and TTMP are shown with dotted line.


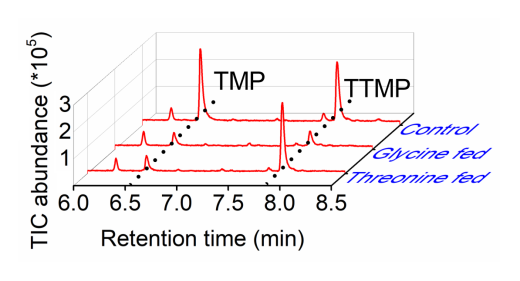


**Fig. S7 TMP and TTMP identification in threonine (glycine) fed *B. dorsalis*.** Traces for TMP and TTMP are shown with dotted line.

**
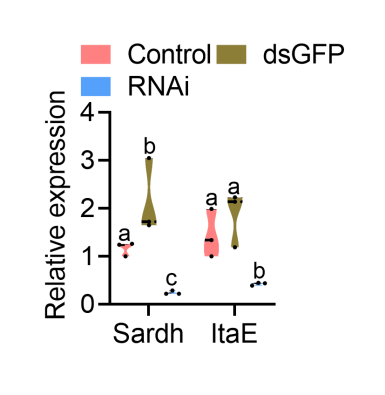
**

**Fig. S8 RNAi effects on *Sardh* and *ItaE* expression in *B. dorsalis*** (*n* = 4 replicates, Different letters indicated significant difference at the 0.05 level, the data was analyzed by ANOVA followed by Tukey’s test).


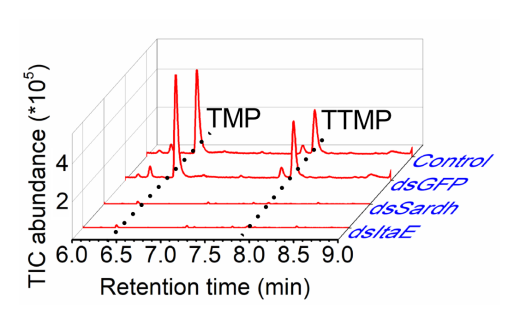


**Fig. S9 TMP and TTMP identification in *B. dorsalis* with *Sardh* and *ItaE* knocked down.** Traces for TMP and TTMP are shown with dotted line.


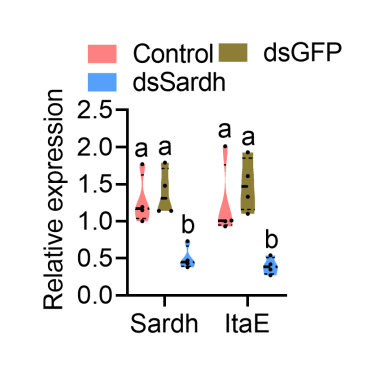


**Fig. S10 RNAi effects on *Sardh* and *ItaE* expression in *B. cucurbitae*** (*n* = 4 replicates, Different letters indicated significant difference at the 0.05 level, the data was analyzed by ANOVA followed by Tukey’s test).

**
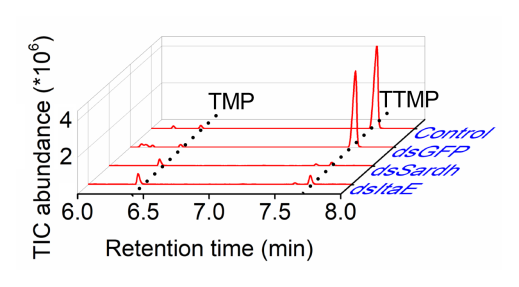
**

**Fig. S11 TMP and TTMP identification in *B. cucurebitae* with *Sardh* and *ItaE* knocked down.** Traces for TMP and TTMP are shown with dotted line.

**
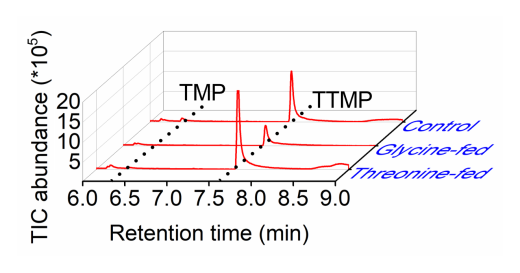
**

**Fig. S12 TMP and TTMP identification in threonine (glycine) fed *B. cucurebitae*.** Traces for TMP and TTMP are shown with dotted line.

**Table S1 Primers used in the study**

| Primer name | Sequence (5’-3’) | Length(bp) |
| --- | --- | --- |
| BD-Sardh-F | CGAAGTCGCGACATACTCCA | 241 |
| BD-Sardh -R | TTCGCACACCATCGGGTTTA |  |
| BD-ItaE-F | GTCCACACTTGCGTCTGTGA | 163 |
| BD-ItaE-R | ACTTTACTATCGTTCGACTCGGAT |  |
| BC-Sardh-F | GCAGTGTCAAGGCAGAGAGT | 207 |
| BC-Sardh -R | CATCGTTGGGTCGTAAACGC |  |
| BC-ItaE-F | ACGCCAAAAGTGCCAATGTC | 206 |
| BC-ItaE-R | GAATTCCCAATCGCGTGAGC |  |
| BC-dsItaE-F | GGATCCTAATACGACTCACTATAAAATGCGCCAAGCCATGTTT | 453 |
| BC-dsItaE-R | GGATCCTAATACGACTCACTATAGCTGTTTGTCACCGTTCACC |  |
| BC-dsSardh-F | GGATCCTAATACGACTCACTATAGACGAGCCTCACGTCAAAG | 219 |
| BC-dsSardh -R | GGATCCTAATACGACTCACTATATGCCGGCTGTTAGTTTAGCA |  |
| BD-dsSardh-F | GGATCCTAATACGACTCACTATATCGCACAGAATCGTGACACA | 288 |
| BD-dsSardh -R | GGATCCTAATACGACTCACTATACCTCGAGCGCTTCTTCGTAA |  |
| BD-dsItaE-F | GGATCCTAATACGACTCACTATAAAAGGAGGCAGGTCTGTTCG | 311 |
| BD-dsItaE-R | GGATCCTAATACGACTCACTATAACTCCAGCGGAATCACCTTG |  |

Note: The red sequence is the T7 promoter.
